# Working memory network plasticity after anterior temporal lobe resection: a longitudinal functional magnetic resonance imaging study

**DOI:** 10.1093/brain/awu061

**Published:** 2014-03-29

**Authors:** Jason Stretton, Meneka K. Sidhu, Gavin P. Winston, Philippa Bartlett, Andrew W. McEvoy, Mark R. Symms, Matthias J. Koepp, Pamela J. Thompson, John S. Duncan

**Affiliations:** 1 Department of Clinical and Experimental Epilepsy, UCL Institute of Neurology, Queen Square, London WC1N 3BG, UK; 2 Department of Neurosurgery, National Hospital for Neurology and Neurosurgery, Queen Square, London WC1N 3BG, UK

**Keywords:** working memory, temporal lobe surgery, epilepsy, functional MRI

## Abstract

Working memory is a crucial cognitive function that is disrupted in temporal lobe epilepsy. It is unclear whether this impairment is a consequence of temporal lobe involvement in working memory processes or due to seizure spread to extratemporal eloquent cortex. Anterior temporal lobe resection controls seizures in 50–80% of patients with drug-resistant temporal lobe epilepsy and the effect of surgery on working memory are poorly understood both at a behavioural and neural level. We investigated the impact of temporal lobe resection on the efficiency and functional anatomy of working memory networks. We studied 33 patients with unilateral medial temporal lobe epilepsy (16 left) before, 3 and 12 months after anterior temporal lobe resection. Fifteen healthy control subjects were also assessed in parallel. All subjects had neuropsychological testing and performed a visuospatial working memory functional magnetic resonance imaging paradigm on these three separate occasions. Changes in activation and deactivation patterns were modelled individually and compared between groups. Changes in task performance were included as regressors of interest to assess the efficiency of changes in the networks. Left and right temporal lobe epilepsy patients were impaired on preoperative measures of working memory compared to controls. Working memory performance did not decline following left or right temporal lobe resection, but improved at 3 and 12 months following left and, to a lesser extent, following right anterior temporal lobe resection. After left anterior temporal lobe resection, improved performance correlated with greater deactivation of the left hippocampal remnant and the contralateral right hippocampus. There was a failure of increased deactivation of the left hippocampal remnant at 3 months after left temporal lobe resection compared to control subjects, which had normalized 12 months after surgery. Following right anterior temporal lobe resection there was a progressive increase of activation in the right superior parietal lobe at 3 and 12 months after surgery. There was greater deactivation of the right hippocampal remnant compared to controls between 3 and 12 months after right anterior temporal lobe resection that was associated with lesser improvement in task performance. Working memory improved after anterior temporal lobe resection, particularly following left-sided resections. Postoperative working memory was reliant on the functional capacity of the hippocampal remnant and, following left resections, the functional reserve of the right hippocampus. These data suggest that working memory following temporal lobe resection is dependent on the engagement of the posterior medial temporal lobes and eloquent cortex.

## Abstract (precis)

Temporal lobe surgery can control seizures in drug-resistant epilepsy, but its impact on working memory is poorly understood. Using functional MRI, Stretton *et al*. reveal improvements in working memory post-surgery, which depend upon the functional capacity of the hippocampal remnant and the functional reserve of the contralateral hippocampus.

## Introduction

Working memory refers to the temporary storage and manipulation of information, and requires continuous updating. Impaired function can disrupt a range of cognitive processes and in consequence, can have a marked impact on everyday activities. Although working memory is generally considered to rely primarily on a frontoparietal network (Cave and Squire, 1992), there is recent evidence that the temporal lobe has an important role in specific aspects of working memory, such as short term maintenance (Axmacher *et al.*, 2007, 2008, 2009; Cashdollar *et al.*, 2009), spatial navigation (Kravitz *et al.*, 2011), and evidence of working memory disruption in temporal lobe epilepsy (TLE) (Owen *et al.*, 1996; Krauss *et al.*, 1997; Abrahams *et al.*, 1999; Wagner *et al.*, 2009; Campo *et al.*, 2012, 2013; Stretton *et al.*, 2012).

Anterior temporal lobe resection (ATLR) is an effective treatment for drug-resistant TLE (Wiebe *et al.*, 2001) rendering 50–80% of patients seizure-free (de Tisi *et al.*, 2011). The effects of ATLR on memory and language functions have been well studied. After left ATLR patients typically show verbal memory decline (Hermann *et al.*, 1995; Sabsevitz *et al.*, 2001; Gleissner *et al.*, 2002, 2004; Bonelli *et al.*, 2010) and less consistently, non-verbal memory decline following right-sided resections (Gleissner *et al.*, 1998; Lee *et al.*, 2002). Surgery involving the language dominant hemisphere is associated with a decline in naming ability (Bonelli *et al.*, 2012; Ives-Deliperi and Butler, 2012).

Frontal lobe function after ATLR has received relatively limited attention and the literature provides mixed results. Improvements in Wisconsin Card Sorting Task performance have been reported in relation to seizure reduction/cessation after ATLR (Hermann *et al.*, 1988; Hermann and Seidenberg, 1995). Other studies however, have reported no significant change in performance after surgery (Trenerry and Jack, 1994; Martin *et al.*, 1999, 2000*a*, *b*). Using a continuous updating word span task Frisk and Milner (1990) found a transient decrease of working memory 3–5 days following surgery but by 2 weeks performance returned to preoperative levels.

It is controversial whether working memory impairment in TLE is a result of temporal lobe dysfunction or is secondary to propagation of epileptic activity to eloquent frontal and parietal cortex (Devinsky, 2005). If medial temporal lobe structures are critical for working memory, then preoperative impairments would be expected and these would persist following surgery (Corcoran and Upton, 1993). If, however, the spread of epileptic activity to eloquent frontoparietal regions underlies working memory difficulties, then removal of the epileptic focus may result in a gain of function (Hermann *et al.*, 1988).

Meta-analyses of ‘*n*-back’ working memory paradigms in healthy volunteers have shown bilateral activation of frontoparietal networks (Owen *et al.*, 2005). Using a similar functional MRI paradigm, we recently found in healthy volunteers that the bilateral medial temporal lobes progressively deactivate in response to increasing working memory load. Conversely, in patients with left and right TLE with hippocampal sclerosis the ipsilesional hippocampus failed to deactivate, and showed aberrant connectivity within the ipsilateral superior parietal lobe. This related to poorer working memory capacity, suggesting that suppression of the hippocampus is required for successful working memory. In parallel, the same patients with TLE showed ipsilesional reduction in superior parietal lobe activation compared with control subjects, suggesting an additional extratemporal dysfunction (Stretton *et al.*, 2012, 2013).

Functional MRI may be used to investigate the extent to which the brain can functionally recover and reorganize following epilepsy surgery. Postoperative functional MRI follow-up studies have the potential to determine the functional capacity of the posterior hippocampal remnant and to evaluate the effectiveness of extratemporal network recovery. We used a visuospatial working memory functional MRI paradigm to investigate: (i) postoperative recovery and reorganization of working memory networks in patients with left and right TLE before and 3 and 12 months after ATLR compared with healthy control subjects; and (ii) the efficiency of these postoperative changes in working memory networks.

## Materials and methods

### Subjects

We studied 33 patients with medically refractory TLE [16 left (eight females); median age 31.5 years, range 19–48 years; 17 right (12 females), median age 40 years, range 18–56 years]. All underwent surgery at the National Hospital for Neurology and Neurosurgery, London.

All patients underwent detailed presurgical evaluation including structural MRI at 3T with qualitative assessment and quantification of hippocampal volumes and T_2_ relaxation times (Woermann *et al.*, 1998; Bartlett *et al.*, 2007), prolonged interictal and ictal video-EEG monitoring and standardized neuropsychological and psychiatric assessments. Structural MRI of the left TLE group identified unilateral hippocampal sclerosis (11 patients), dysembryoplastic neuroepithelial tumours (two patients), cavernoma (one patient), and normal imaging in two patients. In the right TLE group, 10 patients had unilateral hippocampal sclerosis, three dysembryoplastic neuroepithelial tumours, and four had normal imaging. All patients had normal contralateral medial temporal lobe structures on qualitative and quantitative MRI (Woermann *et al.*, 1998). Video- or intracranial-EEG confirmed ipsilateral seizure onset in all patients.

All patients were native English speakers. Handedness was determined using a standardized questionnaire (Oldfield, 1971). All patients underwent working memory functional MRI and standard neuropsychological assessment preoperatively and again at 3 and 12 months postoperatively. All patients were treated with anti-epileptic medication and postoperative changes in medications were recorded (Table 1).

**Table 1:** Clinical characteristics and group demographics

	Controls (*n = *15)	Left TLE (*n = *16)	Right TLE (*n = *17)
Median age (IQR) (years)	40 (30–49)	31.5 (26–45)	40 (22–48)
Handedness (L/R)	4/11	3/13	3/14
Gender (M/F)	9/6	8/8	5/12
Median age at onset (IQR) (years)	n/a	13.5 (4–24)	14.5 (7–33)
Median epilepsy duration (IQR) (years)	n/a	14 (9–18)	18 (6.5–35.5)
AED changes at 3 m	n/a	Nil	Nil
AED changes at 12 m	n/a	2/16 AED free	2/17 AED free
		6/16 One AED reduced	7/17 One AED reduced
ILAE seizure outcome Classification at 3 months	n/a	13/16 Grade 1–2	16/17 Grade 1–2
		3/16 Grade 4–5	1/17 Grade 4
ILAE seizure outcome Classification at 12 months	n/a	14/16 Grade 1–2	14/17 Grade 1
		2/16 Grade 4	3/17 Grade 3–4

AED = Anti-epileptic drugs; ILAE = International League Against Epilepsy; IQR = interquartile range.

The standard neurosurgical procedure was removal of the temporal pole, opening of the temporal horn, followed by *en bloc* resection of the hippocampus with a posterior resection margin at the mid-brainstem level. Postoperative seizure outcome was classified according to the International League Against Epilepsy classification (ILAE) (Wieser *et al.*, 2001) (Table 1). The 3-month postoperative classifications for left TLE were: *n* = 11 grade 1, *n* = 2 grade 2, *n* = 1 grade 4, *n* = 2 grade 5; and for right TLE; *n* = 14 grade 1, *n* = 2 grade 2 and *n* = 1 grade 4. The classifications for left TLE at 12 month after surgery were: *n* = 11 grade 1, *n* = 3 grade 2 and *n* = 2 grade 4; and for right TLE: *n* = 14 grade 1, *n* = 1 grade 3 and *n* = 2 grade 4.

Fifteen native English speaking healthy volunteers (six female; median age 40, range 22–53 years) without any history of neurological or psychiatric disease were assessed on three occasions at ∼6-monthly intervals.

The study was approved by the National Hospital for Neurology and Neurosurgery and the Institute of Neurology Joint Research Ethics Committee, and written informed consent was obtained from all subjects.

### Magnetic resonance data acquisition

MRI studies were performed on a 3 T General Electric Excite HDx scanner. Standard imaging gradients with a maximum strength of 40 mTm^−1^ and slew rate 150 Tm^−1^s^−1^ were used. All data were acquired using an eight-channel array head coil for reception and the body coil for transmission.

For the functional MRI task, gradient-echo planar T_2_*-weighted images were acquired, providing blood oxygenation level-dependent contrast. Each volume comprised 50 oblique axial 2.4 mm slices (with 0.1 mm gap) covering the whole brain, with a 24-cm field of view, SENSE factor 2, 64 × 64 matrix, and an in-plane resolution of 3.75 × 3.75 mm. Echo time was 25 ms, and repetition time was 2.5 s.

### Hippocampal volume measurement

Hippocampal segmentation was performed using MReg; an image registration software package developed in-house (Lemieux *et al.*, 2000). Measurements were derived from a fast inversion recovery prepared spoiled gradient echo sequence T_1_-weighted volumetric (inversion time/repetition time/echo time) 450/8/3 ms, flip angle: 20°, matrix size: 256 × 192, 24 × 18 cm field of view, 124 1.1-mm thick coronal oblique slices. A manual tracing method was employed using a mouse driven cursor.

Preoperatively the posterior boundary of the hippocampus was defined as the oblique coronal section in which the fornix was seen in its fullest profile. The medial limit was taken as the open end of the hippocampal fissure in the middle and posterior portions, and the uncal fissure was used as the boundary anteriorly. The white matter of the temporal stem and/or CSF in the temporal horn provided the lateral limit. The head of the hippocampus was distinguished from the overlying amygdala by the presence of the alveus or uncal recess (Cook *et al.*, 1992).

For postoperative hippocampal volumes the same boundaries were followed where present. The size of the hippocampal remnant varied considerably from patient to patient; if three or more 1.1-mm thick slices were visible the remnant was considered large enough to measure. Measurement started posteriorly, on the oblique coronal section in which the greatest length of fornix was visible and proceeded anteriorly, measuring each contiguous 1.1 mm slice for the remaining postoperative remnant. A standard practice was adopted by both raters, using the manual tracing method; a magnification factor of four was used. The cursor was placed at the most medial, inferior section of the tail, the tracing continued in a clockwise direction for the right hippocampus and anticlockwise for the left. The volume of the region of interest was calculated by multiplying the voxel volume (mm^3^) by the number of screen pixels (at the current degree of magnification) contained within the trace, divided by the square of the magnification factor.

### Neuropsychological assessment

Three working memory span tests were administered to each subject outside of the scanner on each occasion. Span tasks were selected as they require the continuous updating of working memory, are sensitive to the effects of increasing working memory load, and have been shown to be reliant on the frontal lobes (Owen, 2000).

#### Digit Span Backwards

The Digit Span subtest from the Wechsler Adult Intelligence Scale-III (Wechsler, 1997) was administered to each participant and the Digit Span Backwards trials were used as the measure of working memory. The participants have to repeat digit strings of increasing length in the reverse order. Digit sequences ranged from two to eight with two trials per sequence. Span size was calculated as the highest digit sequence where both trials were successful (maximum score = 8).

#### Gesture Span

The Gesture Span task (Canavan *et al.*, 1989) requires the subject to copy sequences of hand gestures of increasing length up to five gestures. The test ends when participants make two consecutive errors at any given gesture set size or when the maximum of five gestures had been reached. The task was repeated with a parallel version immediately after the first version was finished. One point was given for each successful trial. The mean span was calculated across trials and was used for the subsequent analysis (maximum score = 5). Parallel versions of the task were used at each time point.

#### Motor Sequences

The Motor Sequences task devised by Canavan *et al.* (1989) requires a sequence of three hand gestures to be repeated in the same order. Ten alternating sequences were administered in total. The test stopped after all 10 trials had been completed. One point was given for each successful trial. The total number of successful trials was used for the subsequent analysis (maximum score = 10). Parallel versions of the task were used at each time point.

Data were entered into a 3 × 3 repeated measures mixed ANOVA (MANOVA) with factors of Time (three levels) and Group (three levels) to assess main effects and interactions on performance measures and were reported as significant at *P < *0.05 level. Neuropsychological data were analysed using SPSS (v.18).

### Working memory functional magnetic resonance imaging paradigm and data analysis

#### Dot-Back paradigm

**Figure 1:**
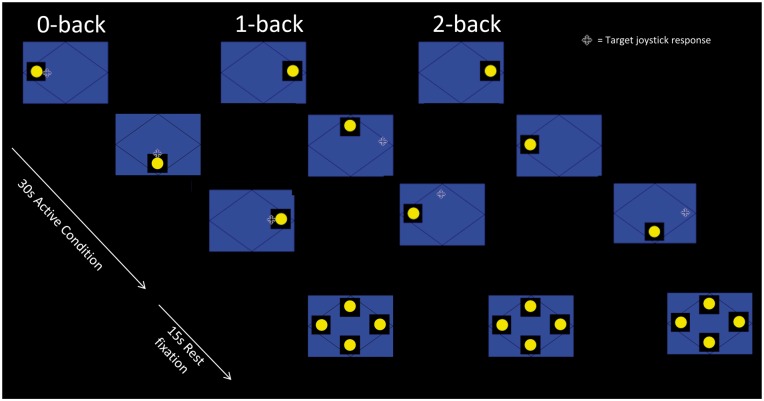
Schematic of the dot-back working memory paradigm. Subjects were asked to monitor the locations of dots within a diamond shaped box on the screen at a given delay from the original occurrence. There were three 30-s active conditions in total (0-, 1-, and 2-back) presented to subjects five times in pseudorandom order and each active condition started with a 15-s resting baseline.

The same working memory paradigm was administered at each assessment (Stretton *et al.* 2012). Subjects were asked to monitor the locations of dots (presentation time: 440 ms; interstimulus interval: 1500 ms) within a diamond-shaped box on the screen at a given delay from the original occurrence (0-, 1-, or 2-back) (Fig. 1). There were three 30-s active conditions in total (0-, 1-, and 2-back) presented to subjects five times in pseudorandom order, controlling for any order effect. In total, 15 stimuli were presented in each 30-s active block. Each active condition started with a 15-s resting baseline (the word ‘Rest’ appeared on the screen during this period). Subjects were asked to move the joystick corresponding to the correct location of the current (0-back) or previously presented (1-back = previous presentation; 2-back = previous presentation but one) stimulus (chance performance = 25%). On-line accuracy data were determined by joystick movement on every trial with output stating either a correct response, wrong response or no response.

#### Data analysis

Imaging data were analysed using Statistical Parametric Mapping (SPM8) (Wellcome Trust Centre for Neuroimaging http://www.fil.ion.ucl.ac.uk/spm). The baseline imaging time series of each subject was realigned using the mean image as a reference. For the two postoperative sessions, following realignment to the mean image as reference, rigid body coregistration was used to coregister postoperative scans to the preoperative mean image; scans were then spatially normalized into standard space applying each subject’s preoperative spatial normalization parameters to the subject’s postoperative realigned and coregistered scans. Preoperatively, a scanner and acquisition specific template created from 30 healthy control subjects, 15 patients with left and 15 patients with right hippocampal sclerosis was used for normalization. All scans were then smoothed with a Gaussian kernel of 8 mm full-width at half-maximum.

At the first level, for each subject, session and trial-specific responses were modelled by convolving a delta function that indicated each block onset with the canonical haemodynamic response function to create regressors of interest, one regressor for each block (‘0-back’, ‘1-back’ and ‘2-back’) in each session. Each subject’s realignment parameters were modelled as confounds for each session, and parameter estimates pertaining to the height of the haemodynamic response function for each regressor of interest were calculated for each voxel. For the baseline session, contrast images for the main effect of multiple-item working memory, ‘2-back’ minus ‘0-back’ and single-item working memory, ‘1-back’ minus ‘0-back’ were created for each subject. One further contrast image was created to model areas of increasingly negative blood oxygen level-dependent signal change in response to increasing task demand to observe progressive deactivation (Vollmar *et al.*, 2011). Rest was modelled implicitly, and the ‘0-back’ condition was used for task-baseline comparison. This condition does not require the manipulation of information within working memory yet controls for visual attention and movement related activity. These contrasts were taken to a second level ANOVA to establish group effects at baseline.

#### Within-subject change over time

To quantify individual changes in functional MRI activation from before to after ATLR in each condition, for each subject, contrast images were created assessing greater/lesser activation and deactivation across the three sessions in each of the three conditions (0, 1 and 2 back). This was achieved using a subtraction contrast, for example, ‘2 back postoperative 3 months’ minus ‘2 back preoperative’ or ‘1 back postoperative 12 months’ minus ‘1 back postoperative 3 months’. This resulted in a set of nine difference contrasts per subject; three contrasts pertaining to changes in 0-, 1-, and 2-back activity between baseline and postoperative 3 months, three pertaining to changes between baseline and postoperative 12 months, and three pertaining to changes between 12 months and 3 months postoperatively. These ‘difference’ contrast images modelled the within subject variance over time in each condition and were taken to second level flexible factorial analyses.

#### Flexible factorial designs

To investigate the relationship between pre- and postoperative change in working memory functional MRI activation and change in performance in each group from before to after ATLR, we used a series of mixed ANOVAs using a flexible factorial design (Gläscher and Gitelman, 2008). This model was applied to each session comparison of interest; postoperative 3 months versus preoperative, postoperative 12 months versus preoperative and postoperative 12 months versus postoperative 3 months. These session comparisons were made between controls and patients with left TLE and controls and patients with right TLE separately owing to the limitation of the flexible factorial only allowing a maximum of two groups to be compared at a time, thus six flexible factorials were run using SPM8. For each flexible factorial, a factor of Group with two levels (controls and patients with left or right TLE) and a factor of Condition with three levels (change of activity in 0, 1, and 2 back) was specified. The three ‘difference’ contrast images pertaining to the session comparison of interest for each subject for each of the three conditions were entered allowing the investigation of a Group × Condition interaction for the contrasts of interest. The resulting matrix generated six regressors of interest, one each for change in 0-, 1-, and 2-back activations for control subjects and the same for the patient group (Supplementary Fig. 1). Time × Group interaction contrasts were then generated pertaining to the contrasts of interest i.e. change in 2-0 back (Gläscher and Gitelman, 2008).

To assess the efficiency of postoperative working memory networks, the individual percentage change in performance for each of the functional MRI conditions (0-, 1- and 2-back) were entered as continuous regressors of interest in each of the corresponding flexible factorials and specified to interact with the factor Group. Contrasts were then generated to examine areas of activity related to changes in performance for each group. Conjunction contrasts were generated *post hoc* to establish whether or not the activity related to change in performance was statistically equivalent to the changes in activity observed between the groups in the contrasts of interest. In addition, to elucidate which conditional (0-, 1- or 2-back) change in performance was associated with the change in activation, the parameter estimates of the peak voxel of the cluster of interest was extracted providing condition-specific values (*n* = 3; one for each of change in 0-, 1-, and 2-back) for each subject. These values were correlated with the corresponding subject-specific changes in performance for each condition using Pearson’s *r* bivariate correlational analysis (two-tailed). To control for differences in baseline performance levels, performance from the first scan for each condition was included as a regressor of no interest for each subject. The out-of-scanner working memory scores were not included as regressors because of insufficient data points per subject as the flexible factorial design matrix requires three values per subject to match the three contrast images entered for the 0-, 1- and 2-back conditions.

We investigated: (i) effects of anterior temporal lobe resection on the functional anatomy of working memory by comparing post- versus preoperative main effects in patients with left and right TLE compared to control subjects; and (ii) efficiency of reorganization of postoperative working memory function by correlating change in activations with postoperative change in dot-back scores in patients with left and right TLE.

To quantify changes in posterior remnant signal, *post hoc* region of interest analysis was performed to extract the mean % blood oxygenation level-dependent signal change in the ipsilateral remnant of the left and right TLE groups, respectively and compared to controls. A 6-mm sphere region of interest (Maldjian *et al.*, 2003) was created for each group centred at *x* = −20, *y* = −33, *z* = −3 for the left hippocampus (based on the peak voxel in the left posterior hippocampus baseline ANOVA) for the left TLE group and at the anatomically homologous region (*x* = 20, *y* = −33, *z* = −3) for the right hippocampus in the right TLE group. Mean % blood oxygenation level-dependent signal changes of these regions were extracted (Brett *et al.*, 2002) at baseline, together with the change in signal at the subsequent time points relative to baseline for each condition of the dot-back task and for each group.

Unless otherwise stated we report all medial temporal lobe activations at a threshold of *P < *0.05, corrected for multiple comparisons (family-wise error in a small volume of interest). In view of our *a priori* hypothesis regarding the hippocampal remnant, coupled with a low signal-to-noise ratio, we performed the small volume correction using a sphere of 6-mm diameter for the left and right hippocampi based on the peak activation in the contrast of interest.

## Results

### Behavioural measures

#### In-scanner performance

There was a significant main effect of Time [*F*(2,90) = 3.422, *P < *0.05] and Group [*F*(2,45) = 3.949, *P < *0.05] on dot-back performance. Overall performance was significantly better at Scan 3 compared with Scan 1 (*P = *0*.*029) irrespective of group. The controls performed significantly better than patients with right TLE irrespective of time (*P = *0*.*029). Increasing working memory load was associated with poorer performance, across the groups [*F*(2,90) = 47.761, *P < *0.05]. The left TLE group significantly improved in 0-back [*t*(14) = 2.122, *P < *0.05] with moderate effect size (*d* = 0.49) and 1-back [*t*(14) = −2.647, *P < *0.05] with large effect size (*d* = 0.89) at 12 months after surgery compared to preoperative levels. Patients with right TLE showed a trend to improve in the same conditions 12 months after surgery. Performance of the 2-back condition was less good than controls for both left and right TLE but it was postoperatively stable (Table 2).

**Table 2:** Group mean behavioural data for each time point

Test	Controls			Left TLE			Right TLE		
	T1, Mean (SD)	T2, Mean (SD)	T3, Mean (SD)	T1, Mean (SD)	T2, Mean (SD)	T3, Mean (SD)	T1, Mean (SD)	T2, Mean (SD)	T3, Mean (SD)
0-Back	92.60 (8.06)	93.20 (7.18)	92.00 (9.47)	80.25 (25.12)	85.63 (25.69)	93.75 (7.81)	75.12 (26.28)	85.18 (16.69)	86.47 (11.86)
1-Back	85.87 (14.93)	87.93 (11.91)	83.27 (19.90)	66.19 (26.81)	73.13 (29.07)	84.19 (13.41)	68.41 (24.31)	77.47 (23.96)	78.00 (22.45)
2-Back	76.93 (18.58)	75.27 (18.01)	76.27 (19.27)	61.25 (21.6)	62.12 (24.43)	64.19 (23.87)	56.00 (23.5)	57.65 (23.41)	58.65 (19.35)
Gesture Span	3.20 (.67)	3.76 (.88)	3.83 (.61)	3.06 (.62)	3.43 (.79)	3.06 (.92)	2.67 (.52)	2.52 (.59)	2.73 (.73)
Motor Sequences	6.60 (1.84)	6.70 (2.12)	7.50 (1.64)	5.25 (1.87)	6.06 (2.20)	5.12 (2.72)	4.29 (2.56)	4.47 (2.09)	4.80 (2.62)
Digit Span Backwards	5.00 (1.31)	5.30 (1.49)	5.06 (1.43)	3.68 (1.07)	3.81 (.65)	3.62 (1.02)	3.52 (.94)	3.64 (.86)	3.52 (1.01)

#### Out-of-scanner performance

There was a significant main effect of Group (all *P < *0.05) on each of the out-of-scanner working memory measures, in that controls performed significantly better than patients with left and right TLE irrespective of time. The main effect of time was not significant on any measure (Table 2).

### Hippocampal volumes

At 3 months after surgery, the left TLE group had significantly (two sample *t*-test, *t* = 2.64, df = 31, *P* = 0.014) larger postoperative residual posterior hippocampal volumes (mean = 76 cm^3^) than the right TLE group (mean = 21 cm^3^). The left TLE group showed significant shrinkage (mean = 0.099 cm^3^, SD = 0.113) of the hippocampal remnant from 3 to 12 months (paired sample *t*-test, *t* = 3.03, df = 31, *P* = 0.011). This effect was not observed in right TLE.

### Functional magnetic resonance imaging

#### Baseline

**Figure 2:**
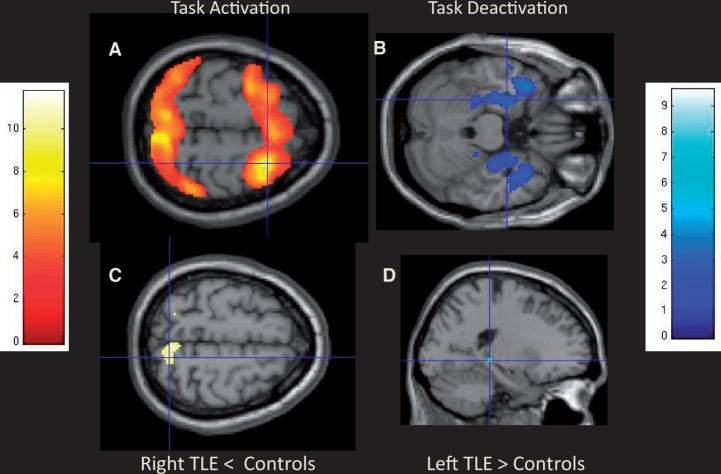
Baseline main effects and between group differences on the dot-back task. Main effects of the 2-0 back contrast show typical working memory fronto-parietal activation (**A**) and progressive deactivation of the default mode network including the medial temporal lobes (**B**) across all groups. Controls activated the right superior parietal lobes significantly more than the right TLE group (**C**), whereas the left TLE group deactivated the posterior left hippocampus significantly more than the control group (**D**).

At baseline, all three groups showed typical frontoparietal activation during working memory conditions with stronger effects occurring with increasing task demands. Both left and right TLE groups had significantly reduced activation of the bilateral superior parietal lobes (*P < *0.001 uncorrected) with stronger effects occurring with increasing task demands (Fig. 2). All three groups showed progressive deactivation of the typical default mode network including bilateral precuneus, medial frontal gyrus, inferior parietal lobes and mesial temporal lobe. In patients, the ipsilateral anterior hippocampus failed to deactivate. Patients with left TLE progressively deactivated the right anterior hippocampus and left posterior hippocampus (*P < *0.005 uncorrected) significantly more than controls with increasing task demand (Fig. 2). There were no other significant differences in progressive deactivation between controls and right TLE.

#### Within-group change in control subjects

There were no significant differences in the task positive working memory networks across all time points. There was greater progressive deactivation of default mode network with increased task demands, including the left and right hippocampus, at Scan 2 compared with Scan 1. On Scan 3 there was greater deactivation of the medial frontal gyrus and right hippocampus than on Scan 1, but not Scan 2.

### Differences between preoperative and 3 months postoperative scans

For details see Supplementary Table 1.

#### Left temporal lobe epilepsy versus control subjects

The left TLE group showed no significant change in task-positive working memory network activations at 3 months after surgery, compared to controls. However, the progressive deactivation of the default mode network with increased task demands showed significant changes compared with control subjects.

Relative to controls, the left TLE group did not increase the deactivation of four regions with increased task demands: left posterior middle temporal gyrus, left precuneus, left posterior hippocampus and right anterior hippocampus (Supplementary Fig. 2).

#### Efficiency of 3-month postoperative change in left temporal lobe epilepsy

**Figure 3:**
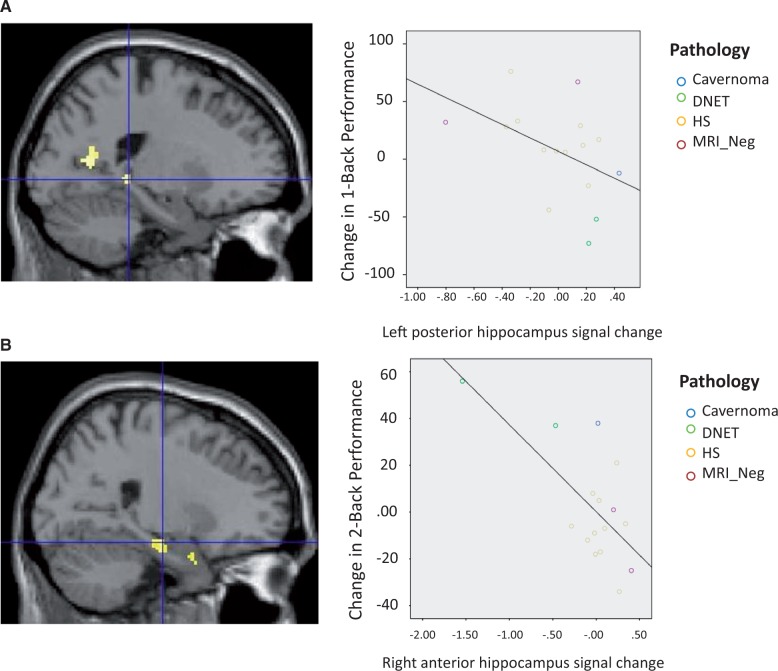
Main between-group differences following ATLR and correlation with performance. Patients with left TLE showed failure to increase deactivation of the left posterior hippocampus 3 months after surgery compared to baseline, which was related to greater 1-back performance (**A**). Between 3 and 12 months after surgery, the left TLE group showed increased deactivation of the contralateral right anterior hippocampus compared to controls correlating with greater improvement in 2-back performance (**B**). DNET = Dysembryoplastic neuroepithelial tumour; HS = hippocampal sclerosis.

A conjunction analysis between less improvement in left TLE dot-back performance from Scan 1 to Scan 2 and left TLE < controls deactivations revealed both the left (−22, −32, −6; z = 2.04, *P = *0.021) (Fig. 3A) and right posterior hippocampus (20, −30, −4; 2.28, *P = *0.011), indicating that failure to increase the deactivation of these regions with increased task demands was associated with less improvement in postoperative dot-back performance. Correlation analysis revealed that this was specific to the change in 1-dot-back performance (r = −0.457, *P* < 0.0.05) rather than 0- (r = −0.159, *P = *0.278) and 2-back (r = −0.209, *P = *0.219) performance (Fig. 3A).

#### Right temporal lobe epilepsy versus control subjects

At 3 months postoperatively the right TLE group showed significant increases in the right superior parietal lobe activation compared to controls during 2-0 back (Supplementary Fig. 3). This region activated less in the right TLE group preoperatively than the control subjects. Between the baseline and 3 months postoperatively, the right TLE group did not increase the deactivation of the left inferior parietal lobe or the right inferior parietal lobe to the extent that was seen in the controls.

#### Efficiency of 3-month postoperative change in right temporal lobe epilepsy

There were no correlations between changes in activation/deactivation and dot-back performance.

### Differences between preoperative and 12 months postoperative working memory activations

For details see Supplementary Table 2.

#### Left temporal lobe epilepsy versus control subjects

There were no significant changes between controls and the left TLE group in the task-positive working memory network or progressive deactivation of the default mode network 12 months after surgery compared to baseline. The failure of increased deactivation observed 3 months after surgery in the left posterior middle temporal gyrus, left precuneus and left posterior hippocampus was no longer apparent.

#### Efficiency of 12-month postoperative change in left temporal lobe epilepsy

Greater improvement in dot-back performance at Scan 3 compared to Scan 1 was associated with increased deactivation of the left posterior hippocampus (−32, −38, 0; *z* = 2.66, *P = *0.05 small volume correction 6 mm sphere). Correlation analysis revealed this was not specific to any condition (all *P* > 0.05).

#### Right temporal lobe epilepsy versus control subjects

At a less conservative threshold, at 12 months the right TLE group activated the right superior parietal lobe more than preoperatively compared with controls during 2-0 back. There was no significant difference in the change of progressive deactivation of the default mode network compared with controls. The failure of increased deactivation observed at 3 months after surgery in the bilateral inferior parietal lobes was no longer apparent.

#### Efficiency of 12 month postoperative change in right temporal lobe epilepsy

There were no correlations between changes in activation/deactivation and dot-back performance.

### Differences between postoperative 12 month and postoperative 3 month working memory activations

For details see Supplementary Table 3.

### 

#### Left temporal lobe epilepsy versus controls subjects

Between 3 and 12 months postoperatively, the left TLE group showed greater progressive deactivation with increased task demands of the right hippocampus compared with controls (Supplementary Fig. 4). There was no significant difference in task-positive working memory network activation.

### Efficiency of postoperative change between 3 and 12 months in left temporal lobe epilepsy

Conjunction analysis revealed greater improvement in dot-back performance was associated with the increased deactivation of the right anterior hippocampus (26, −16, −18; *z* = 2.11, *P < *0.05 small volume correction 6 mm) (Fig. 3B), an effect that was not evident on comparison with preoperative data. Correlation analysis revealed this was related to improvement in 0-back (r = −0.666, *P* < 0.005), 1-back (r = −0.837 *P* < 0.001) and 2-back (r = −0.684, *P* < 0.005) performance.

#### Right temporal lobe epilepsy versus controls subjects

Compared to controls, the right TLE group showed greater progressive deactivation of the right posterior hippocampus 12 months postoperatively compared with 3 months.

### Efficiency of postoperative change between 3 and 12 months in right temporal lobe epilepsy

Conjunction analysis revealed a trend toward greater deactivation of the right posterior hippocampus (26, −32, 4, *z* = 1.94, *P = *0.09 small volume correction 6 mm) was associated with less improvement in dot-back performance. Correlational analysis showed this relationship was not specific to any condition (all *P* > 0.05).

#### Change in posterior hippocampal remnant signal over time

**Figure 4:**
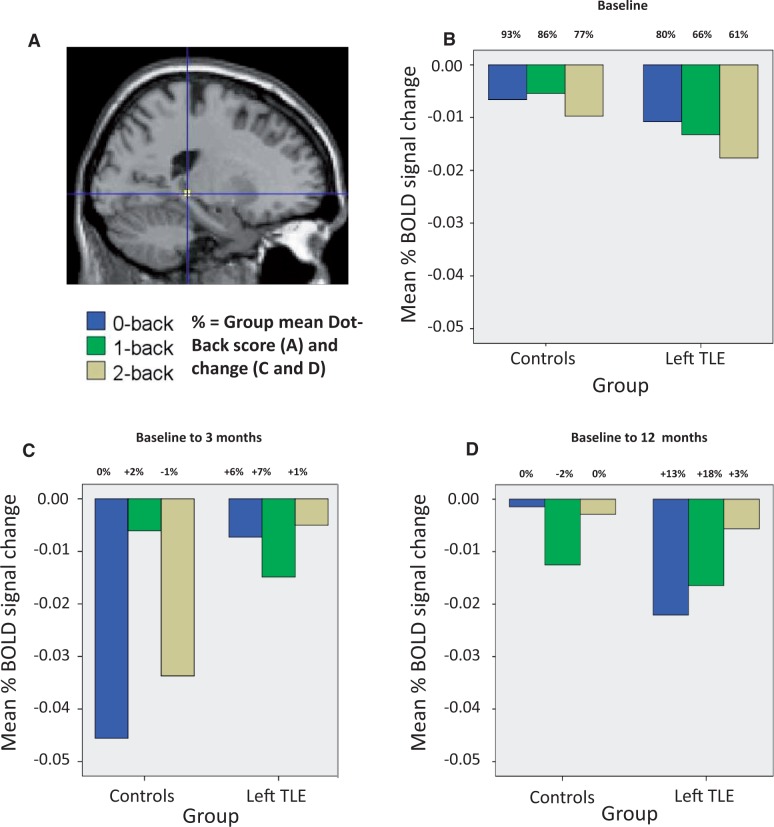
Mean % blood oxygenation level-dependent signal change in the left posterior hippocampus (**A**) at baseline assessment (**B**), 3 months after surgery compared to baseline (**C**), and at 12 months after surgery compared to baseline (**D**) relative to controls. Presented at the top of the bars are the group mean baseline dot-back for each condition (**B**) and subsequent group mean % changes in performance relative to time point (**C** and **D**). BOLD = blood oxygen level-dependent.

**Figure 5:**
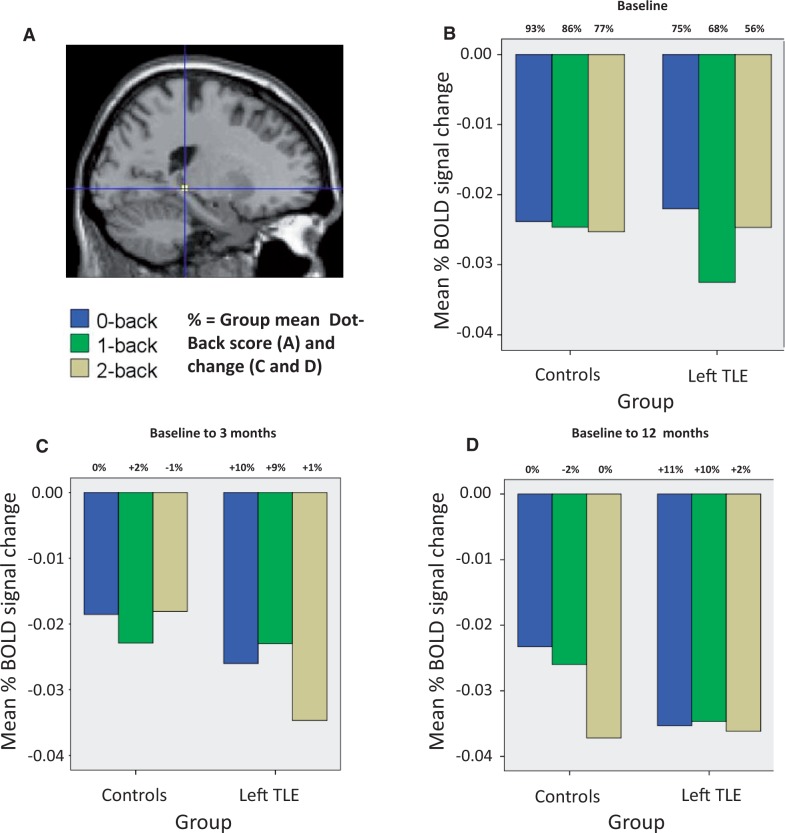
Mean % blood oxygenation level-dependent signal change in the right posterior hippocampus (**A**) at baseline assessment (**B**), 3 months after surgery compared to baseline (C), and at 12 months after surgery compared to baseline (**D**) relative to controls. Presented at the top of the bars are the group mean baseline dot-back for each condition (**A**) and subsequent group mean % changes in performance relative to time point (**C** and **D**). BOLD = blood oxygen level-dependent.

To quantify further and visualize the changes observed in the ipsilateral posterior hippocampal remnant signal, a *post hoc* region of interest analysis was performed. In the left TLE group (Fig. 4), analysis confirmed greater progressive deactivation of the left posterior hippocampus in the left TLE group at baseline relative to controls (Fig. 4B). Three months after surgery compared with baseline, the left TLE group failed to increase the deactivation of this region compared to controls (Fig. 4C); however, at 12 months after surgery compared to baseline the left posterior hippocampus deactivated significantly more than controls (Fig. 4D), particularly for the 0 and 1-back conditions which correlated with improved performance (see above and Fig. 3). In the right TLE group (Fig. 5), analysis confirmed no baseline differences in signal change of the right posterior hippocampus between controls and right TLE (Fig. 5B). At 3 (Fig. 5C) and 12 (Fig. 5D) months after surgery both groups increased the deactivation of this region but in the right TLE group was associated with less improvement in performance (see above and Fig. 3).

## Discussion

At baseline all three groups showed typical working memory activation patterns of the fronto-parietal network and deactivation of the default mode network, including the hippocampus bilaterally in control subjects, though only contralateral to the epileptic focus in patients with TLE. Individuals with left TLE had impaired working memory performance compared with controls and showed reduced activation of the bilateral parietal lobes and greater deactivation of the left posterior and right anterior hippocampus. Individuals with right TLE were also impaired relative to controls on working memory measures and showed reduced bilateral superior parietal lobe activation as previously reported (Stretton *et al.*, 2012). In the controls some changes in deactivation patterns were observed over three scans over 12 months while performance remained stable. In comparison to controls, patients with left and right TLE showed significantly different changes in activations/deactivations and performance over these intervals after ATLR:

- Left TLE patients, and to a lesser degree right TLE, progressively improved attention (0-back) and single-item (1-back) working memory at 3 and then 12 months after surgery. There was no decline on any working memory measures.
- At 3 months after surgery compared to baseline, patients with left TLE failed to increase deactivation of the hippocampal remnant and this was related to lesser improvement in task performance. Patients with right TLE showed increased activation of the right superior parietal.
- At 12 months after surgery compared to baseline, patients with left TLE showed increased deactivation of the hippocampal remnant and this was related to improved performance. Patients with right TLE again showed increased right superior parietal lobe activation.
- Between 3 and 12 months after surgery, patients with left TLE showed increased deactivation of the right anterior hippocampus and this was related to improved performance. Patients with right TLE showed increased deactivation of the right posterior hippocampus though this was related to less improvement in task performance.

### Comparison to previous findings

Previous neuropsychological and imaging studies have shown working memory impairments in patients with both left and right TLE (Owen *et al.*, 1996; Krauss *et al.*, 1997; Abrahams *et al.*, 1999; Wagner *et al.*, 2009; Campo *et al.*, 2012, 2013; Stretton *et al.*, 2012, 2013). A systematic review (Sherman *et al.*, 2011) showed few studies had evaluated executive function as part of a pre/post-surgery study design (Davies *et al.*, 1998; Martin *et al.*, 2000*a*; Helmstaedter *et al.*, 2003), with only one examining working memory as part of an attention task. These studies found no adverse effect of epilepsy surgery, with mild gains of function reported in verbal fluency and attention following both left and right ATLR (Sherman *et al.*, 2011). Additionally, one longitudinal study assessed verbal working memory span in the immediate (3–5 days) and early (2 weeks) postoperative recovery phase (Frisk and Milner, 1990) after left ATLR. Patients showed a transient decline in the immediate phase before recovery at 2 weeks, returning to baseline levels, with no effect of the extent of hippocampal excision (Frisk and Milner, 1990). Although such early postoperative assessment was likely inadequate to capture plasticity and the longer-term cognitive trajectories of these processes, the failure to observe persistent decline is of note. Our results complement and extend these findings, in that both left and right TLE groups showed no decline in both in- and out-of-scanner measures of working memory 12 months after surgery. We also observed improvements in attention (0-back condition) and single-item working memory (1-back condition) in both patient groups and this was more prominent in the left TLE group who performed at control levels 12 months after surgery. Improvements in attention after ATLR have been previously reported (Helmstaedter *et al.*, 2003), and are thought to reflect the reduction/cessation of seizure activity following surgery, with little comment on the contribution of the hippocampus to attentional processing. However, it is important to note, and of potential interest for future studies, that greater hippocampal deactivation in this condition correlated with improved 0-back performance, indicating a potential functional role for the hippocampus in visuospatial attentional processing. The less marked improvement in the right TLE group may reflect the visuospatial nature of the functional MRI task employed with greater sensitivity to right hemispheric/hippocampal function. This was evidenced by significantly increased deactivation of the right hippocampus between scans in our control group. Although this could indicate material-specificity of working memory, no significant between-group differences were observed between left or right groups on task performance, or on the verbal (digit span) and non-verbal (gesture span, motor sequences) working memory measures. This supports the notion that low-level working memory demands can be supported in the absence of one anterior temporal lobe, yet higher working memory load likely requires the support of bilateral medial temporal lobe structures for optimal performance. This is supported by the results of the 2-back performance over time, as scores at 3 and 12 months following unilateral ATLR remained at baseline level. Similarly, the capacity of both verbal and non-verbal working memory span outside of the scanner was maintained for lower demand (∼3 digits on the Digit Span task and ∼3 gestures on the Gesture Span task) for both groups yet remained poorer than control subjects in which bilateral mesial temporal lobe structures remained intact. Future studies to address the role of the uni- and bilateral contribution to low and high working memory load are warranted.

Furthermore, we suggest the differential neural trajectory between the left and right TLE groups in the presence of similar behavioural trajectory may be due to the baseline activation differences observed on the task. Subjects with right TLE showed a relatively hypoactivated right superior parietal lobe compared with controls, thus a greater variation in the signal of this region is likely to be unearthed relative to controls who showed little variation of signal in this region. As the left TLE group showed no such preoperative hypoactivation, a similar trajectory of right superior parietal activation is assumed relative to controls. On the contrary, the left TLE group showed increased deactivation of the left posterior hippocampus at baseline whereas the right TLE did not. Thus, again, a wider variation of change in signal magnitude could be observed in the controls group relative to the left TLE group (i.e. increase deactivation from Time 1 to Time 2). Overall, in the context of ILAE class 1–2 seizure outcomes in 85% of patients in this study, our findings support the hypothesis that removal of an epileptic focus and of epileptic activity spreading to eloquent cortex can underpin postoperative improvement in single-item working memory 12 months after surgery (Hermann *et al.*, 1988).

The few longitudinal studies that have employed cognitive functional MRI paradigms before and after ATLR have investigated the reorganization of language (Wong *et al.*, 2009; Bonelli *et al.*, 2012) and episodic memory networks (Maccotta *et al.*, 2007; Cheung *et al.*, 2009; Bonelli *et al.*, 2013). None of these included a longitudinal control group. Our findings provide evidence for the complexity of neural functional plasticity as it evolves during the first postoperative year and demonstrate different patterns dependent on the laterality of the resection. Shortly following a left temporal lobe resection we observed a failure to increase engagement of the posterior hippocampal remnant and the greater the failure, the poorer the performance. This supports our recent finding of a positive correlation of working memory performance and preoperative posterior hippocampal deactivation (Stretton *et al.*, 2012) and the importance of the change in left posterior hippocampus activation in supporting verbal episodic memory before and early after ATLR (Bonelli *et al.*, 2013). However, these changes were not static and at 12 months the left posterior hippocampal remnant showed greater deactivation as compared to preoperative baseline, mirroring the deactivation pattern of the control group.

For the right-sided resections at 3 months there was no support for the importance of the right hippocampal remnant but we did observe increased activation of the ipsilateral superior parietal lobe. Previous studies have reported cortical reorganization of language function (Bonelli *et al.*, 2012) and reductions of activity within the frontal lobes (Maccotta *et al.*, 2007; Wong *et al.*, 2009). However, we did not observe any significant performance correlation with the increase in right superior parietal lobe activity in the right TLE group, which limits the interpretation of this as evidence that the removal of the epileptic right anterior temporal lobe released preoperatively suppressed function of the right superior parietal lobe. It is possible that 12 months after ATLR remains too early for the distant parietal cortex to become functionally incorporated into the working memory network.

Similar to the left-sided resections, we observed a delayed recovery of the right hippocampal remnant showing greater deactivation at 12 months compared with 3 months relative to controls although this was related to less improvement in performance. In addition, although both patient groups showed increased deactivation of the posterior hippocampus 12 months after surgery compared to baseline, the left TLE group showed greater working memory gains than the right TLE group. Furthermore, patients with left TLE showed greater preoperative deactivation of the posterior hippocampus compared to controls whereas the right TLE group did not. This indicates that preoperative compensation may be more beneficial than postoperative compensation in supporting working memory. This is in keeping with recent work showing that postoperative reorganization to the hippocampal remnant was associated with poorer memory outcome (Bonelli *et al.*, 2013). Our study provides evidence that in a working memory task that requires bilateral mesial temporal lobe recruitment, the functional capacity of the posterior to-be-resected hippocampus helps support postoperative working memory.

### Neurobiological and clinical implications

#### Compensatory mechanisms

Between 3 and 12 months after left-sided surgery the deactivation of the right hippocampus remained significantly greater than controls and only between these time points became an efficient process, correlating with improved performance. This important finding provides evidence for functional compensation of the contralateral hippocampus, and raises the possibility that the effectiveness of compensation is a dynamic process related to the length of time after surgery. Combined with the results of previous studies (Cheung *et al.*, 2009; Bonelli *et al.*, 2013), our data suggest that 3 months after surgery may be too early in the postoperative recovery phase to adequately capture neural evidence of functional compensation of the contralateral medial temporal lobe.

#### The role of the hippocampus in working memory

Whether the hippocampus is part of the neural system subserving working memory is the subject of debate (Ezzyat and Olson, 2008; Baddeley *et al.*, 2011). It has been proposed as a candidate structure for the episodic buffer that provides an interface between verbal and non-verbal working memory subsystems and a link to longer term stores (Hannula *et al.*, 2006; Olson *et al.*, 2006; Ezzyat and Olson, 2008). We previously reported that during continuous updating of working memory with increasing cognitive load the hippocampi progressively deactivated to maintain optimal performance and were functionally connected to the task-specific default mode network (Stretton *et al.*, 2012, 2013). After ATLR with removal of the diseased anterior hippocampus, working memory performance did not decline in either patient group, a finding that would seem to argue against critical hippocampal involvement in working memory. We did, however, find postoperative changes in hippocampal activation patterns that related to working memory efficiency indicating a role for these structures in working memory processing. These findings suggest the continuous updating of visuospatial working memory, which typically requires the bilateral suppression of the hippocampi, can be supported by the unilateral suppression of a healthy anterior temporal lobe after the removal of an epileptogenic one.

#### Clinical implications

Working memory did not decline following ATLR irrespective of side or extent of resection. We cannot comment as to whether the effect is directly due to seizure freedom as we had too few patients with continued seizures to make a comparison with those who were seizure-free. However, reanalysis of the data with non-seizure free patients removed made no significant differences to either the functional MRI or the behavioural results.

### Strengths and limitations

A major advantage of this study is the repeated measures design with the inclusion of healthy control subjects scanned on three occasions. This design has the advantage of controlling for within-group changes of the functional MRI signal over time, test–retest reliability and practice effects. It also allows direct statistical comparison of between-group changes whilst controlling for within-subject and within-group changes. A further advantage is the 3 and 12 month time-points after surgery, allows us to probe the temporal patterns of recovery from surgery.

Limitations were imposed by the clinical nature of the study; principally there was a variable interval between the baseline scan and surgery. The average interval between pre- and 3 month postoperative scans was 8 months for left TLE, and 13 months for right TLE. The controls averaged 6 months between Scans 1 and 2. However, the intervals between Scans 2 and 3 were matched in all groups. We noted significant differences in hippocampal remnant volume between patient groups which could influence the functional MRI signal in this region. However, there was no correlation between postoperative remnant volume and functional MRI signal of the remnant, indicating the changes in this region are more related to function rather than structure. Furthermore, although all patients underwent the same surgery, and the majority of patients had hippocampal sclerosis, the underlying pathologies were heterogeneous across the groups, which could significantly impact the interpretation of the results. Studies of larger cohorts of each pathological group examining this issue are desirable for future research. Additionally, by 12 months after surgery several patients had changed their anti-epileptic medication dosages, with a small number having discontinued their medications. This effect was not controlled for in the functional MRI analyses; however, drug reduction patterns were similar for both patient groups (7/16 left TLE; 8/17 right TLE). Nevertheless, the potential influence of anti-epileptic medication on cognition and functional MRI signal cannot be ignored (Kim *et al.*, 2006; Yasuda *et al.*, 2013). Longer follow-up studies of seizure- and drug-free patients would allow the delineation of these effects. Finally, changes in mood have not been accounted for in the current study. Depression and anxiety can increase after ATLR (Foong and Flugel, 2007), and there is a limited understanding of the relationship between epilepsy and affective disorder and how this relationship is influenced by ATLR.

In conclusion, we have shown that working memory-related mesial temporal lobe network plasticity is differentially affected after left or right ATLR for TLE. Appropriate engagement of the hippocampal remnant is required for successful visuospatial working memory recovery and maintenance. Following right ATLR, reorganization to the hippocampal remnant between 3 and 12 months postoperatively was not associated with improved efficiency. Following left ATLR, the contralateral hippocampus becomes efficient in parallel with normalization of the remnant function between 3 and 12 months after surgery.

## Supplementary Material

Supplementary Data

## Abbreviations

ATLR: anterior temporal lobe resection
TLE: temporal lobe epilepsy
