# Supplementary material for: Working memory network plasticity after anterior temporal lobe resection: a longitudinal functional magnetic resonance imaging study
Source: Brain. 2014 Mar 29;137(5):1439–53. doi: 10.1093/brain/awu061 (PMC3999723; doi:10.1093/brain/awu061)
Supplement: Supplementary Data [file supp_137_5_1439__index.html]

Working memory network plasticity after anterior temporal lobe resection: a longitudinal functional magnetic resonance imaging study — Supplementary Data 

# Working memory network plasticity after anterior temporal lobe resection: a longitudinal functional magnetic resonance imaging study

## Supplementary Data

files

**Files in this Data Supplement:**

- Supplementary Data - docx file
