# Supplementary material for: Working memory network plasticity after anterior temporal lobe resection: a longitudinal functional magnetic resonance imaging study
Source: Brain. 2014 Mar 29;137(5):1439–53. doi: 10.1093/brain/awu061 (PMC3999723; doi:10.1093/brain/awu061)
Supplement: Supplementary Data [file supp_awu061_brain-2013-00739-File009.docx]

**Supplementary Material**

**Working memory network plasticity following anterior temporal lobe resection: a longitudinal functional magnetic resonance imaging study.**

J Stretton^1^**,** MK Sidhu^1^, GP Winston^1^, P Bartlett^1^, AW McEvoy**^2^**, MR Symms**^1^**, MJ Koepp**^1^**, PJ Thompson**^1^_,_** JS Duncan**^1^**

**^1^ Epilepsy Society MRI Unit, Epilepsy Society, Chalfont St Peter, SL9 0RJ**

**Department of Clinical and Experimental Epilepsy, UCL Institute of Neurology,**

**Queen Square, London WC1N 3BG, UK.**

**and**

**^2^Department of Neurosurgery, National Hospital for Neurology and Neurosurgery, Queen Square, London WC1N 3BG, UK.**

**Figure S1. Schematic of the data analytic strategy employed.** At the first level, difference contrasts were generated for each subject for each condition (0, 1 and 2 back) over, for example, two time points. These contrasts were inputted in to a second level flexible factorial design with group and condition as factors. The allowed the investigation of the interaction between group and condition whilst implicitly modelling the factor of time.


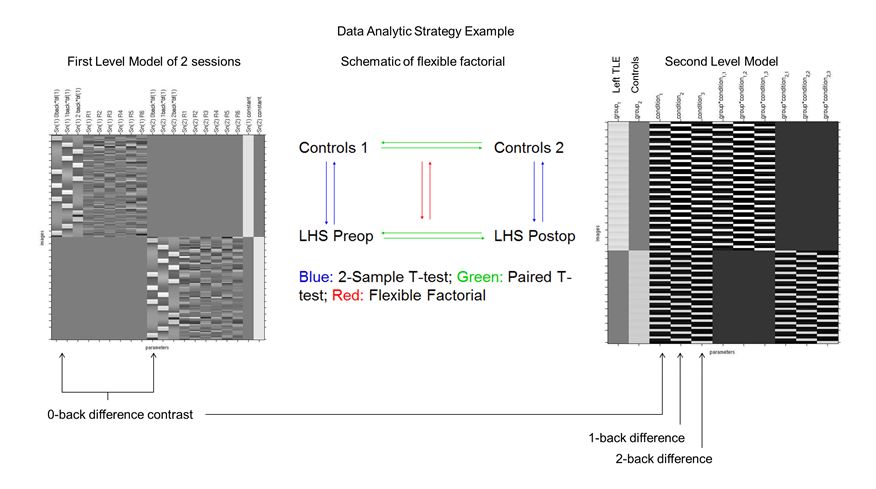


**Supplementary Figure S2: Differences between preoperative and 3 months postoperative scans**; **Controls > Left TLE.** The left TLE group failed to increase the deactivation of 4 regions with increased task demands: left posterior middle temporal gyrus, left precuneus, left posterior hippocampus (not shown) and right anterior hippocampus (not shown)


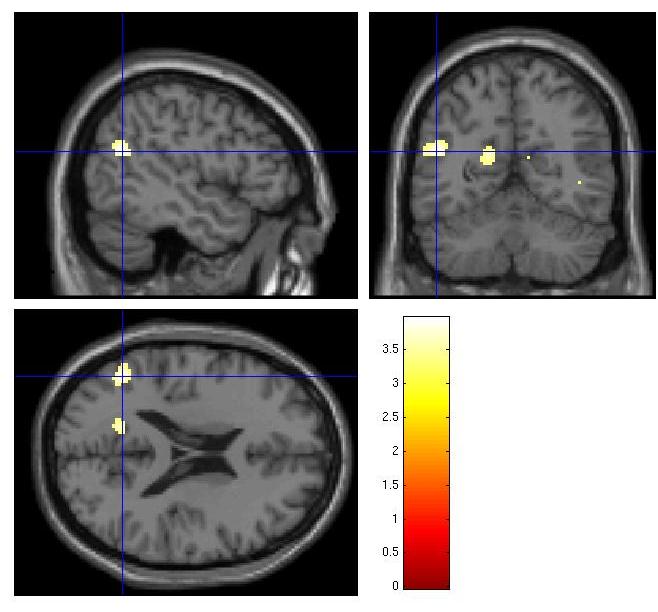


**Supplementary Figure S3: Differences between preoperative and 3 months postoperative scans**; **Right TLE > Controls.** The right TLE group showed significant increases in the right superior parietal lobe activation compared to controls during 2-0 back


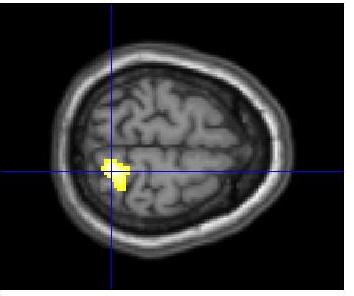

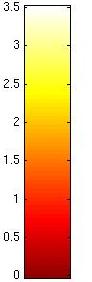


**Supplementary Figure S4: Differences between postoperative 12m and postoperative 3m WM activations; Left TLE > Controls.** The left TLE group showed greater progressive deactivation with increased task demands of the right hippocampus compared to controls


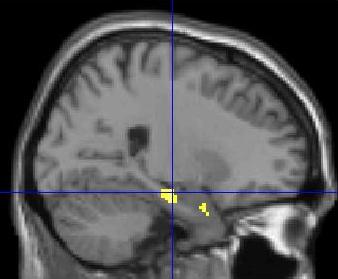

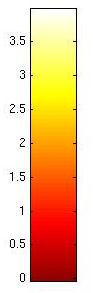


**Supplementary Figure S5: Differences between postoperative 12m and postoperative 3m WM activations; Right TLE > Controls.** Compared to controls, the right TLE group showed greater progressive deactivation of the right posterior hippocampus 12 months postoperatively compared to 3 months


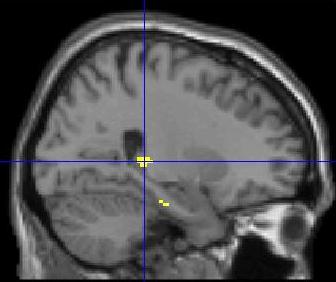

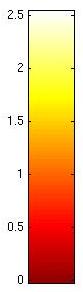


**Supplementary Table 1.** Differences in change of group activation at 3 months postoperative

| Interaction | Contrast | Region | MNI Coordinates (x, y, z) | Z Score | P value (unc.) |
| --- | --- | --- | --- | --- | --- |
| Controls > Left TLE | 2-0 | - | - | - | - |
|  | 1-0 | - | - | - | - |
|  | Progressive deactivation | L Middle Temporal Gyrus | -48, -58, 20 | 3.79 | 0.001 |
|  |  | L Precuneus | -12, -56, 18 | 3.62 | 0.001 |
|  |  | L Hippocampus | -20, -36, -2 | 3.20 | 0.001 |
|  |  | R Hippocampus | 24, -12, -18 | 3.33 | 0.001 |
| Left TLE > Controls | 2-0 | - | - | - | - |
|  | 1-0 | - | - | - | - |
|  | Progressive deactivation | - | - | - | - |
| Controls > Right TLE | 2-0 | - | - | - | - |
|  | 1-0 | - | - | - | - |
|  | Progressive deactivation | L Inferior Parietal Lobe | -52, -58, 26 | 3.45 | 0.001 |
|  |  | R Inferior Parietal Lobe | 48, -60, 34 | 2.78 | 0.005 |
| Right TLE > Controls | 2-0 | R Superior Parietal Lobe | 26, -74, 58 | 2.96 | 0.005 |
|  | 1-0 | - | - | - | - |
|  | Progressive deactivation | - | - | - | - |

**Supplementary Table 2.** Differences in change of group activation at 12 months postoperative

| Interaction | Contrast | Region | MNI Coordinates (x, y, z) | Z Score | P value (unc.) |
| --- | --- | --- | --- | --- | --- |
| Controls > Left TLE | 2-0 | - | - | - | - |
|  | 1-0 | - | - | - | - |
|  | Progressive deactivation | - | - | - | - |
| Left TLE > Controls | 2-0 | - | - | - | - |
|  | 1-0 | - | - | - | - |
|  | Progressive deactivation | - | - | - | - |
| Controls > Right TLE | 2-0 | - | - | - | - |
|  | 1-0 | - | - | - | - |
|  | Progressive deactivation | - | - | - | - |
| Right TLE > Controls | 2-0 | R Superior Parietal Lobe | 16, -56, 68 | 3.60 | 0.01 |
|  | 1-0 | - | - | - | - |
|  | Progressive deactivation | - | - | - | - |

**Supplementary Table 3.** Differences in change of group activation between 3 and 12 months postoperative.

| Interaction | Contrast | Region | MNI Coordinates (x, y, z) | Z Score | P value (unc.) |
| --- | --- | --- | --- | --- | --- |
| Controls > Left TLE | 2-0 | - | - | - | - |
|  | 1-0 | - | - | - | - |
|  | Progressive deactivation | - | - | - | - |
| Left TLE > Controls | 2-0 | - | - | - | - |
|  | 1-0 | - | - | - | - |
|  | Progressive deactivation | R Anterior Hippocampus | 24, -14, -14 | 3.70 | 0.001 |
| Controls > Right TLE | 2-0 | - | - | - | - |
|  | 1-0 | - | - | - | - |
|  | Progressive deactivation | - | - | - | - |
| Right TLE > Controls | 2-0 |  |  |  |  |
|  | 1-0 | - | - | - | - |
|  | Progressive deactivation | R Posterior Hippocampus | 26, -32, 4 | 2.25 | 0.05 (SVC) |
